# Opinion research among Russian Physicians on the application of technologies using artificial intelligence in the field of medicine and health care

**DOI:** 10.1186/s12913-023-09493-6

**Published:** 2023-07-13

**Authors:** I.A. Orlova, Zh.A. Akopyan, A.G. Plisyuk, E.V. Tarasova, E.N. Borisov, G.O. Dolgushin, E.I. Khvatova, M.A. Grigoryan, L.A. Gabbasova, A.A. Kamalov

**Affiliations:** 1 Medical Research and Education Center of Lomonosov, Moscow State University, 27/10 Lomonosov Prospect, Moscow, 119192 Russia; 2 Faculty of Fundamental Medicine, Lomonosov Moscow State University, 27/1 Lomonosov Prospect, Moscow, 119192 Russia

**Keywords:** Artificial intelligence, AI, Awareness, Physicians

## Abstract

**Background:**

To date, no opinion surveys has been conducted among Russian physicians to study their awareness about artificial intelligence. With a survey, we aimed to evaluate the attitudes of stakeholders to the usage of technologies employing AI in the field of medicine and healthcare and identify challenges and perspectives to introducing AI.

**Methods:**

We conducted a 12-question online survey using Google Forms. The survey consisted of questions related to the recognition of AI and attitudes towards it, the direction of development of AI in medicine and the possible risks of using AI in medicine.

**Results:**

301 doctors took part in the survey. 107 (35.6%) responded that they are familiar with AI. The vast majority of participants considered AI useful in the medical field (85%). The advantage of AI was associated with the ability to analyze huge volumes of clinically relevant data in real time (79%). Respondents highlighted areas where AI would be most useful—organizational optimization (74%), biopharmaceutical research (67%), and disease diagnosis (52%). Among the possible problems when using AI, they noted the lack of flexibility and limited application on controversial issues (64% and 60% of respondents). 56% believe that AI decision making will be difficult if inadequate information is presented for analysis. A third of doctors fear that specialists with little experience took part in the development of AI, and 89% of respondents believe that doctors should participate in the development of AI for medicine and healthcare. Only 20 participants (6.6%) responded that they agree that AI can replace them at work. At the same time, 76% of respondents believe that in the future, doctors using AI will replace those who do not.

**Conclusions:**

Russian doctors are for AI in medicine. Most of the respondents believe that AI will not replace them in the future and will become a useful tool. First of all, for optimizing organizational processes, research and diagnostics of diseases.

**Trial registration:**

This study was approved by the Local Ethics Committee of the Lomonosov Moscow State University Medical Research and Education Center (IRB00010587).

**Supplementary Information:**

The online version contains supplementary material available at 10.1186/s12913-023-09493-6.

## Background

Artificial intelligence (AI) is defined as the ability of computer systems to perform tasks that would normally require human-level intelligence. AI technologies are already widely used in the field of computer sciences [1–3], the manufacturing sector, the information sphere and the development of communications [4]. In recent years, the use of AI systems in the field of medicine has been increasingly discussed [5–9]. There have been publications on the successful use of AI systems (IIS) to determine the likelihood of developing genetic variations in low-grade gliomas [10], to identify genetic phenotypes in small cell lung carcinoma [11], to reduce the number of false-positive results in computer screening mammography [12], to detect pathology of lymphatic nodes of the mediastinum [13], and automatic determination of bone age [14]. These examples demonstrate the potential benefits of using AI in medicine and create the preconditions for using AI in other directions in the future. It is obvious that the introduction of AI in medicine and healthcare will lead to changes in the doctors’ work [15].

There are different views on the future of AI. The pessimistic view of AI is that AI will replace humans in many industries. There is also an optimistic view that people will have more opportunities to benefit from clinical advances in the future with the help of AI. However, there have been no studies of the attitude of Russian doctors to the use of artificial intelligence systems in medicine. Medical students and young doctors will obviously come into contact with AI during their career. Therefore, doctors must be prepared for these changes in order to effectively use AI as a useful tool in their work.

The aim of this study was to study the awareness of Russian doctors about AI and assess their opinion on the use of technologies using artificial intelligence in medicine and healthcare.

## Methods

This study was approved by the Local Ethics Committee of the Lomonosov Moscow State University Medical Research and Education Center. Using Google Forms, we interviewed residents and graduate students of medical universities, doctors working in various medical organizations, research centers and universities. The survey was conducted online via an invitation to a mobile phone or email. The selection criteria for the initial invitation included current medical occupation and interest in AI. The response rate wasn’t considered. The necessary information was collected about the personal and professional data of the respondents. Each participant was sent a link to the online survey. Participants were informed about the purpose of the survey (medical research) in the introduction to the questionnaire. The survey was anonymous. The invitation link was generated randomly and wasn’t recorded. When starting the survey, the respondents confirmed their voluntary informed consent. We confirm that participation was voluntary; the participants could not be identified on the basis of submitted materials and could not be harmed as a result of the research. Replies were made on a single web page with a single submit button that only allows submissions to these links.

Our online survey, consisting of 12 closed-ended questions (Additional file 1: Appendix 1), was conducted in December 2021. The questionnaire from the study by Oh, S et al. [16] was taken as a basis adding one extra question regarding the relationship between doctors using AI and those who not. The authors' permission for its translation, adaptation and use was obtained. The validity of the content of the questionnaire was verified by researchers (*n* = 5) and a group of doctors (*n* = 10) by valuing the diagnostic efficiency of the questionnaire on the basis of other existing questionnaires on this topic. The decision on which questionnaire should be taken and adopted or which additional questions might be included was made within the joint committee of the Russian Regenerative Medicine Society and The Russian Heart Failure Society. The questions selected were discussed to be most worrying problems among Russian physicians. The survey consisted of questions about AI awareness, confidence in A, direction of AI in medicine, and the potential risks of using AI.

Answers to seven questions (B1-B7) were assessed using a five-point ordinal Likert scale (from 1 = strongly disagree to 5 = strongly agree). Five possible answers were given to answer three additional questions (B8, B10, B11). Questions B9 and B12, related to taking responsibility for decisions, had 1 answer.

In the first part of the survey, the question was asked about the awareness of doctors towards AI and their opinion about the medical use of AI. The doctors were then asked about the areas of medicine in which it is possible to use AI and what problems they are concerned about regarding the use of AI in medicine. There is a lot of discussion in the media about who is responsible in situations when there are adverse clinical outcomes—AI or human, so we included the question of AI responsibility in medicine.

### Subgroup analysis: age, occupational status and clinical experience

We investigated whether the attitude towards the use of AI in medicine changes depends on the age, professional status, scientific degree and place of work of the respondent. All of the demographics were collected within and at the beginning of the questionnaire.

Categories of professional status: novice doctor (resident or graduate student), clinician, diagnostician. The question about the availability of a scientific degree suggested the answers: graduate students, M.D., Ph.D..

The experience categories were determined by the number of years spent after receiving accreditation: less than 10 years, from 10 to 20 years, and more than 20 years.

The place of work was defined as a scientific and educational institution, a city hospital, a polyclinic, a private clinic, or something else.

### Statistical analysis

Basic statistics (median, lower and upper quartiles, or total and percentages) were calculated for all covariates. In subgroup analyzes, the Kruskal–Wallis tests were used to assess the effect of gender. Differences in response based on work status and clinical experience were analyzed using the Mann–Whitney test. For all tests, the significance level was set at *P* ≤ 0.05.

## Results

The survey was completed by 301 participants, with an average age of 29 (25; 53) years. The average work experience in medicine was 5 (1–27) years. The participants were 143 novice doctors, 44 diagnostic doctors and 114 clinicians. The distribution by specialties was as follows: therapy—65 respondents; cardiology—54 respondents; clinical laboratory—11 respondents; radiology—10 respondents; ultrasound diagnostics—7 respondents; functional diagnostics—9 respondents; urology—8 respondents; psychiatry—6 respondents; obstetrics and gynecology—13 respondents; surgery—15 respondents; ophthalmology—3 respondents; anesthesiology and resuscitation—8 respondents; neurology—11 respondents; traumatology and orthopedics—9 respondents; rheumatology—2 respondents; endocrinology—11 respondents; dentistry—4 respondents; hematology—2 respondents and others—54 respondents. It might be noted that the vast majority of the participants are female as it reflects the general population demographics of Russian doctors.

The characteristics of the participants is presented in Table 1.

**Table 1:** The Characteristics of survey participants

	n/%
*Age*	
< 30 years	139/46,2%
31 – 40	48/16%
41 – 50	36/12.0%
51 – 60	59/19,6%
61 – 70	15/5,0%
> 70	4/1,3%
*Gender*	
Female	216/71,8%
Male	85/28,2%
*Professional status*	
Diagnostician	44/14,6%
Clinician	114/37,9%
Novice doctor (resident or graduate student)	143/47,5%
*Experience in medicine*	
0–10 years	177/58,8%
11–20	27/9,0%
20–30	45/15,0%
31–40	43/14,3%
> 40	9/3,0%
*Scientific degree*	
Graduate students	225/74,8%
M.D	55/18,3%
M.D. Ph.D	21/6,9%
*Place of work*	
Scientific and educational institution	199/66,1%
Polyclinic	23/7,6%
City hospital	40/13,3%
Private clinic	15/5,0%
Other places	24/8,0%

The results of the survey are presented in Tables 2 and 3.

**Table 2:** Doctors’ awareness of AI and their opinion towards its use in medicine and healthcare. Answers to questions (B1-B7), assessed using a five-point ordinal scale (n /%)

	Absolutely agree	Rather agree	Can’t decide	Rather disagree	Absolutely disagree
1. Do you agree that you are familiar with artificial intelligence?	14/4,7%	93/30,9%	43/14,3%	131/43,5%	20/6,6%
2. Do you agree that AI has useful applications in medical field?	132/43,8%	124/41,2%	29/9,6%	15/5,0%	1/0,3%
3. Do you agree that the diagnostic capabilities of AI are superior to the clinical experience of a human physician?	9/3,0%	40/13,3%	63/20,9%	148/49,2%	41/13,6%
4. Do you agree that AI can replace you in your job?	7/2,3%	13/4,3%	19/6,3%	118/39,2%	144/47,8%
5. Do you agree that AI will not replace doctors, but doctors using AI will replace doctors who do not?	81/26,9%	149/49,5%	34/11,3%	30/10,0%	7/2,3%
6. Do you agree that you will always use AI to make medical decisions in the future?	23/7,6%	112/37,2%	80/26,6%	71/23,6	15/5,0%
7. Do you agree that physicians should be involved in the development of AI for health care?	168/55,8%	99/32,9%	22/7,3	7/2,3	5/1,7%

**Table 3:** Doctors’ opinion regarding the directions of using AI in medicine and health care and responsibility for their use. Answers to 8–12 questions

	n/%
8. What are the benefits of using AI? (1 or more statements could be selected)	
AI will be able to optimize organizational processes in healthcare	205/68,1%
AI can help reduce medical errors	157/52,2%
AI can provide massive amounts of clinically relevant, high-quality data in real time (support for physician decisions)	238/79,1%
AI is available anytime, anywhere	147/48,8%
AI is not subject to emotional exhaustion or physical fatigue	215/71,4%
9. If your medical judgment and AI judgment differ, what will you follow? (only 1 statement could be selected)	
Doctor’s opinion	259/86,0%
AI’s opinion	12/4,0%
Patient’s choice	30/10,0%
10. In what area of medicine do you think artificial intelligence will be most useful? (1 or more statements could be selected)	
Establishing diagnosis	154/51,2%
Making treatment decisions	78/25,9%
Actual treatment (including surgery)	51/16,9%
Biopharmaceutical R&D	202/67,1%
Providing medical care in remote areas	138/45,6%
Optimization of organizational decisions	224/74,4%
11. What worries you about the use of AI in medicine? (1 or more statements could be selected)	
It cannot be used in unexpected situations due to inadequate information	169/56,2%
It is not flexible enough to be applied to every patient	192/63,8%
Difficult to apply on controversial issues	182/60,5%
Low ability to empathize and take into account patient's emotional state	15/50,2%
It is developed by specialists with little clinical experience in medicine	100/33,2%
12. Who do you think will be responsible for possible legal problems caused by artificial intelligence? (only 1 statement could be selected)	
Doctor	179/59,5%
The company that created AI	98/31,9%
The patient who agreed to follow AI’s directions	26/8,6%

107 (36%) out of 301 survey participants agree that they are familiar with technologies that use AI (B1, Table 2). 32% of female doctors and 42% of male doctors confirmed that they are familiar with AI. Diagnostic doctors in a larger percentage than clinicians know about AI—45% versus 35%. Novice doctors confidently answered that they were well acquainted with the problem in 32% of cases.

**Fig. 1:**
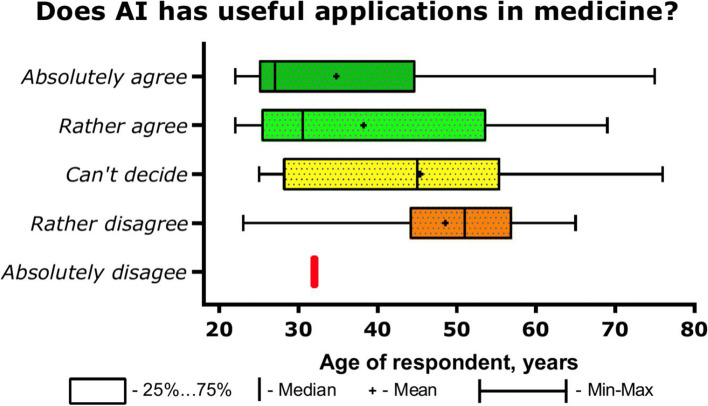
The answer to the question about the potential benefits of AI in medicine, depending on age

At the same time, the overwhelming majority of Russian doctors believe that AI has useful applications in medicine (85%, 256/301) and only 1 respondent answered categorically no (B2, Table 2). There were no significant gender differences in the answer to this question. The answer to the question about the potential benefits of AI in medicine, depending on age, is shown in Fig. 1. From Fig. 1. it can be seen that there was a tendency towards a more pessimistic assessment of the prospects for AI among older doctors. Diagnosticians see possibilities of using AI more clearly, 82% of them believe that AI will be useful in medicine. Clinicians are more restrained, suggesting that AI is beneficial in 74% of cases. The most optimistic are novice doctors, 95% of them believe in the future benefits of AI.

Russian doctors, in general, are not inclined to believe that the diagnostic capabilities of AI are superior to the experience of a human doctor (B3, Table 2). Only 16% of the respondents took the side of the AI. Female doctors speak about the diagnostic priorities of FIS only in 11%, and among male doctors this opinion is shared by a significantly larger number of respondents—31% (*p* < 0.001). On this question, both diagnosticians and clinicians have a consolidated opinion—they agree that the diagnostic capabilities of AI exceed the experience of a human doctor in 20% and 19%, respectively. Doctors of medical sciences (MD) are more likely to side with AI (28%) than candidates of medical sciences (PhD) (15%) and doctors without a degree (7%). There were no significant differences in the age and experience of the respondents.

Concern that AI can replace a doctor at his workplace (B4, Table 2) was expressed only by 20 respondents (6.6%). Both female doctors (91%), male doctors (87%), diagnosticians (82%), and clinicians (85%) do not expect such a development of events. And even novice doctors in 90% of cases believe that AI will not be able to replace them in the future. There were no differences in the answers in the subgroups, divided by age, length of service, and availability of an academic degree.

**Fig. 2:**
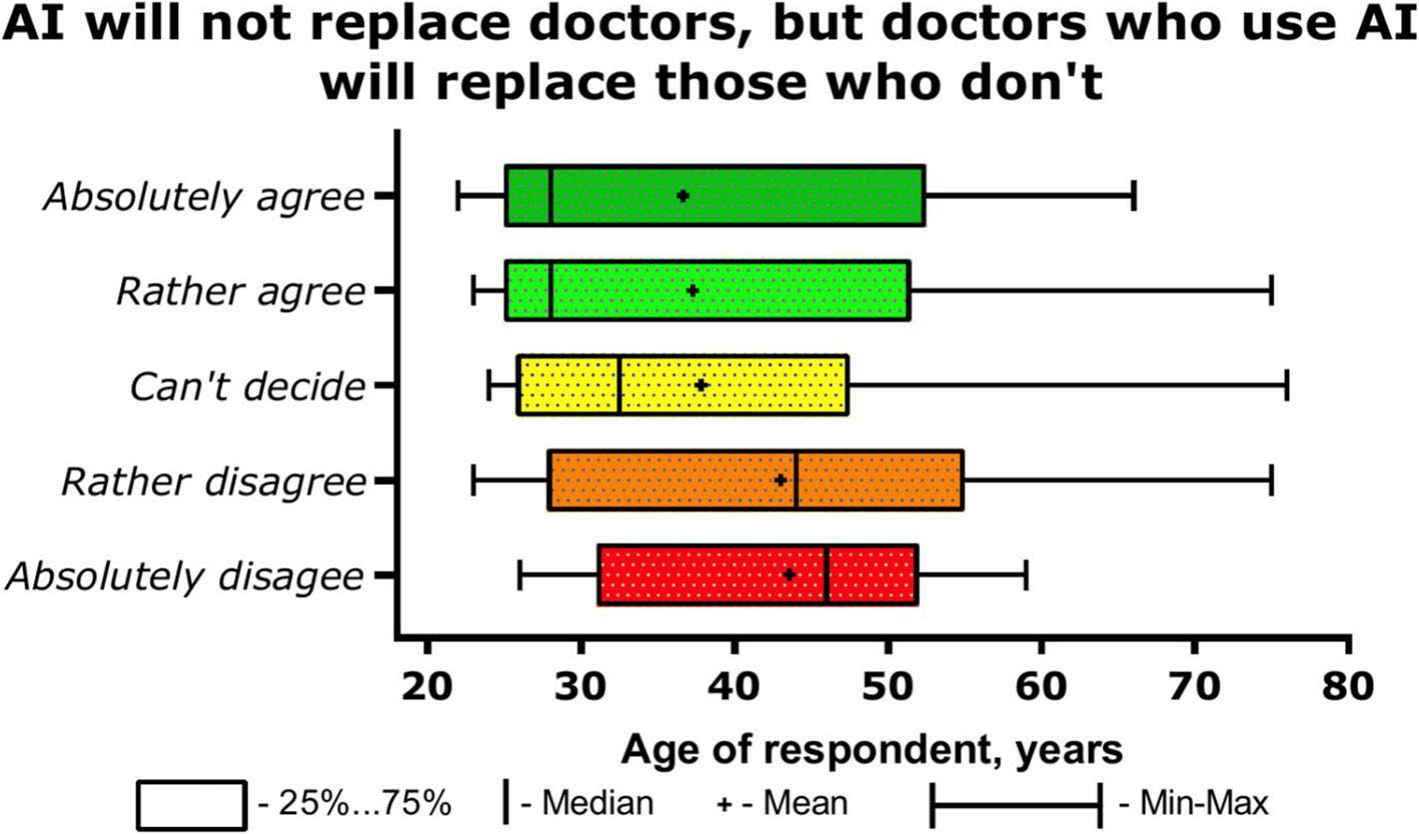
The answer to the question about the potential replacement of doctors using AI with those who do not, depending on the age

At the same time, the overwhelming majority of doctors believe that over time, doctors using AI will replace doctors who do not (B5, Table 2)—230 / 76.5%. This is the opinion of 68% diagnostic doctors, 63% clinicians and 82% novice doctors. 48% MDs, 29% PhDs, 28% of doctors without a degree agree with them. There was no gender difference in the answer to this question. The answer to this question depending on age is shown in Fig. 2.

With regard to the imperative use of AI in making decisions in the future (B6, Table 2), the opinion of Russian doctors was divided. 45% of respondents agree with this prospect, 29% disagree, 26% have not yet decided. There were no differences depending on gender, age, experience and professional status. 63% of M.D.s expected that it would happen, in contrast to the Ph.D.s (47%) and doctors without a degree (43%).

The overwhelming majority of respondents believe that doctors should participate in the process of developing AI for health care (B7, Table 2)—267/89%, and only 12 people believe that this should not be done. When analyzed in subgroups, the answers were evenly distributed.

Advantages of AI are associated with the ability to analyze huge volumes of clinically relevant data in real time (79%). 68% hope for the assistance of AI in optimizing organizational processes in health care. The majority of doctors note the ability to use AI at any time and in any place without fear of its burnout (Table 3).

### Expected areas of application in medicine

The respondents identified areas in which AI would be most useful—optimization of organizational decisions (74%), biopharmaceutical research (67%) and disease diagnosis (52%) (Table 3).

### Potential risks

Among the possible problems in the use of AI, they noted the lack of flexibility and limitation of application on controversial issues (64% and 60% of respondents). 56% believe that AI decision making will be difficult if inadequate information is presented for analysis. A third of doctors fear that specialists with little experience took part in the development of AI, and 89% of respondents believe that doctors should participate in the development of AI for medicine and health (Table 3).

### Responsibility

**Fig. 3:**
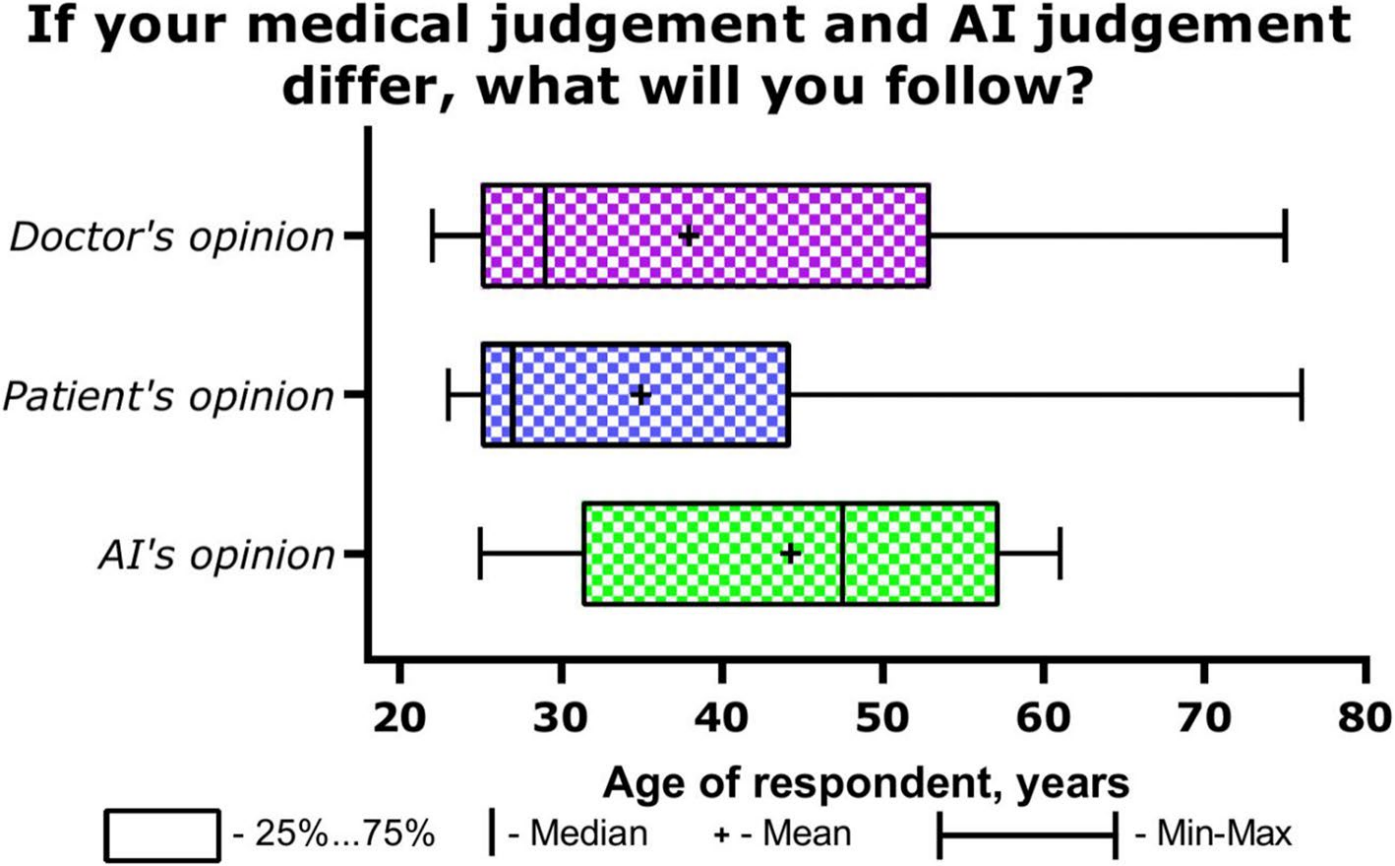
The answer to the question to whose judgment will a doctor follow if his medical judgment and that of artificial intelligence differ, depending on age

When having a controversy with AI, most doctors believe that it is necessary to make a decision, which the doctor suggests—259/86%. The results of this section are presented in Table 3. 30/10% of respondents believe that it is worth giving the choice to the patient and the remaining 12/4% will follow the opinion of AI. Among M.D.s there was not a single person who was ready to prefer the opinion of AI. There were no significant differences depending on gender, age, lexperience and professional status in the answer to this question. However, it is noteworthy that older doctors are more inclined to follow the opinion of AI (Fig. 3).

In case of problems caused by AI, respondents believed that the responsibility should be taken by doctors (59.5%, 179/301), patients who agreed to use AI (8.6%, 26/301) and the company that created AI. (31.9%, 96/301). 66% of clinicians, 59% of diagnosticians and 55% of novice doctors are ready to take responsibility. 32% of clinicians, 25% of diagnosticians and 38% of novice physicians believe that the AI manufacturing company should take responsibility. The most responsible seem to be Ph.D.s. They are ready to accept responsibility in 71% of cases, M.D.s. in 52% and doctors without a degree in 57%. There were no significant differences depending on gender, age, experience and professional status of doctors in the answer to this question.

## Discussion

On the whole we see that the necessity of both evaluation and adoption of AI in the doctors’ everyday practice is of great importance. This may be seen by observing the articles on that subject. Artificial Intelligence in healthcare has great potential to address some of the most significant medical and health challenges. The healthcare industry and patients should benefit from AI development and safe usage [26]. However, its difficult to dismiss the problems of immediate integration of AI into physicians’ medical routine incliding misunderstanding of AI processes, the lack of technical base and mere unwillingness to unite the work with AI [27].

A database search revealed 10 publications devoted to the analysis of doctors' opinions regarding AI [16–25]. As far as we know, our work is the first Russian study to study the attitude of doctors towards AI. Our results showed that a third of the surveyed doctors in the Russian Federation are familiar with AI technologies. This figure is 2 times less than according to a survey of American doctors [20], but 5 times higher than in a Korean study [16]. The overwhelming majority of respondents in our study see useful applications of AI in medicine and healthcare (85%) and believe that in the future, doctors who use AI in their work will replace those who do not (76%). Russian doctors do not express concern about competition with AI, 87% of them do not assume that AI will be able to completely replace them in the foreseeable future. A close attitude to the prospects of using AI in medicine is demonstrated by medical specialists in other countries [19, 22]. In our study, novice doctors and specialists mainly engaged in diagnostics (radiology, laboratory diagnostics) were expected to be more optimistic. We can observe a similar situation in lots of studies including the one concerning the attitude of undergraduate medical students in radiology [25].

79% of physicians thought that the advantage of AI is the ability to analyze huge volumes of clinically relevant data in real time. 68% hope for the assistance of AI in optimizing organizational processes in healthcare. The majority of doctors note the ability to use AI at any time and anywhere without fear of its burnout (Table 3). A similar opinion is expressed by doctors in other studies [16, 25]).

Russian doctors believe that the areas in which AI would be most useful include optimization of organizational decisions (74%), biopharmaceutical research (67%), and disease diagnostics (52%) (Table 3). In a study by Huisman M. et al., which included radiologists from 54 countries [26], 77% of doctors associated the prospects of using AI with the optimization of organizational processes in a medical institution, and 78%—with obtaining a “second opinion”. Neurosurgeons have expressed enthusiasm for the use of AI for image interpretation (62%), operational planning (82%), and surgical team coordination (70%) [21].

In most surveys, ethical and legal issues (62%) and lack of knowledge (57%) were most often mentioned as obstacles to the implementation of AI in real practice [25].

Among the possible problems when using AI, Russian doctors noted the lack of flexibility and limitation of use on controversial issues (64% and 60% of respondents). 56% believe that AI decision making will be difficult if inadequate information is provided for analysis. A third of doctors are afraid that specialists with little experience took part in the development of AI, and 89% of surveyed believe that doctors should be involved in the development of AI for medicine and healthcare.

In cases when opinion of Russian doctors differ from AI’s one, physicians believe that it is necessary to make a decision that the doctor proposes—259/86%, 30/10% of respondents believe that it is worth entrusting the choice to the patient and the remaining 12/4% will follow the opinion of AI (Table 2). Speaking about the possible problems associated with the use of AI, 59.5% of Russian doctors are ready to take responsibility, 31.9% say that it is the responsibility of the company that created AI, and 8.6% believe that the patient can take responsibility by agreeing with AI’s recommendations. In a study by Reffien et al. [25], the results of a survey of Malaysian physicians were very similar. 81.7% of respondents trust the opinion of a doctor, not AI’s. 49.7% believe that the doctor is responsible for AI decisions, 34.7%—the AI manufacturer, 15.6%—the patient who agreed to follow the AI instructions.

### Restrictions

Some limitations of our study should be noted. First, we couldn't ask open-ended questions. All participants could have different conceptual ideas about AI. Secondly, there is a possibility of selection bias. Participants may have been more motivated and may have expressed more positive attitudes than those who did not take part in the survey. Since the data was self-reported, bias cannot be ruled out. Third, the AI questionnaire was designed by physicians, not AI experts.

## Conclusion

This study showed that Russian doctors are generally favorable to the use of AI technologies in medicine and healthcare. Most of the respondents believe that AI will not replace them in the future and will become a useful tool, first of all, for optimizing organizational processes, scientific research and diagnosing diseases.

The analysis made it possible to identify potential vulnerabilities for using AI systems in medicine and healthcare:

- Low level of physicians’ awareness about the possibilities of AI and its potential applications in medicine and healthcare. Two-thirds of Russian doctors who participated in our survey, and among them a quarter had an academic degree and two-thirds worked in scientific and educational institutions, could not say about themselves that they are well acquainted with AI.
- The range of areas in which doctors see real prospects for the use of AI is limited—optimization of organizational decisions (74%), biopharmaceutical research (67%) and diagnosis of diseases (52%).
- Low confidence of doctors in AI: concern about the low quality of data used to train AI, inability to use in controversial issues, and lack of participation of expert doctors in the development of AI. In favor of the cautious attitude of Russian doctors to the use of AI in medicine is the fact that only half of those surveyed suggest that AIS will be able to reduce the number of medical errors. In addition, with a discrepancy between the judgments of a doctor and artificial intelligence, only 4% of doctors are ready to follow the decision of AI.
- Ethical and legal uncertainty regarding the distribution of responsibility when using AI. Currently, in the Russian Federation, technologies using AI have the status of a medical product. So, a priori, the final decision, along with full responsibility, is made by the doctor. At the same time, according to our data, about a third of Russian doctors believe that the company that created the AI should be responsible.

Thus, further research in this area and educational programs based on the data obtained may in the future contribute to greater physician confidence in technologies that use artificial intelligence and the implementation of AI in real clinical practice.

## Supplementary Information

**Additional file 1.**

## Data Availability

The datasets used and/or analyzed during the current study are available from the corresponding author on reasonable request due to privacy of our interviewees.
