# Supplementary material for: Opinion research among Russian Physicians on the application of technologies using artificial intelligence in the field of medicine and health care
Source: BMC Health Serv Res. 2023 Jul 13;23:749. doi: 10.1186/s12913-023-09493-6 (PMC10339534; doi:10.1186/s12913-023-09493-6)
Supplement: Supplementary file 1 — Additional file 1. [file 12913_2023_9493_MOESM1_ESM.docx]

**Annex 1.** Online survey form.

QUESTIONNAIRE

Age_______years, Gender – M/F, Resident/PhD student

clinician, specialty ______________________________________________

Diagnostic doctor: radiology / functional diagnostics / ultrasound diagnostics / other

Work experience in medicine - _____ years, Ph.D. / M.D. / without a degree

Place of work - scientific and educational institution / city hospital / polyclinic / private clinic / other

|  | ***Choose one statement that is the closest to you.*** | | | | |
| --- | --- | --- | --- | --- | --- |
|  | Absolutely agree | Rather agree | Can’t decide | Rather disagree | Absolutely disagree |
| 1. Do you agree that you are familiar with artificial intelligence? |  |  |  |  |  |
| 2. Do you agree that AI has useful applications in medical field? |  |  |  |  |  |
| 3. Do you agree that the diagnostic capabilities of AI are superior to the clinical experience of a human physician? |  |  |  |  |  |
| 4. Do you agree that AI can replace you in your job? |  |  |  |  |  |
| 5. Do you agree that AI will not replace doctors, but doctors using AI will replace doctors who do not? |  |  |  |  |  |
| 6. Do you agree that you will **always** use AI to make medical decisions in the future? |  |  |  |  |  |
| 7. Do you agree that physicians should be involved in the development of AI for health care? |  |  |  |  |  |
| 8. What are the benefits of using AI? (**1 or more statements could be selected**) | | | | | |
| AI will be able to optimize organizational processes in health care | | | | | |
| AI can help reduce medical errors. | | | | | |
| AI can provide massive amounts of clinically relevant, high-quality data in real time (support for physician decisions) | | | | | |
| AI is available anytime, anywhere | | | | | |
| AI is not subject to emotional exhaustion or physical fatigue | | | | | |
| 9. If your medical judgment and AI judgment differ, what will you follow? (**only 1 statement could be selected**) | | | | | |
| Doctor's opinion | | | | | |
| AI’s opinion | | | | | |
| Patient’s choice | | | | | |
| 10. In what area of medicine do you think artificial intelligence will be most useful? (**1 or more statements could be selected**) | | | | | |
| Establishing diagnosis | | | | | |
| Making treatment decisions | | | | | |
| Actual treatment (including surgery) | | | | | |
| Biopharmaceutical R&D | | | | | |
| Providing medical care in remote areas | | | | | |
| Optimization of organizational decisions | | | | | |
| 11. What worries you about the use of AI in medicine? (**1 or more statements could be selected**) | | | | | |
| It cannot be used in unexpected situations due to inadequate information | | | | | |
| It is not flexible enough to be applied to every patient | | | | | |
| Difficult to apply on controversial issues | | | | | |
| Low ability to empathize and take into account patient's emotional state | | | | | |
| It is developed by specialists with little clinical experience in medicine | | | | | |
| 12. Who do you think will be responsible for possible legal problems caused by artificial intelligence? (**only 1 statement could be selected**) | | | | | |
| Responsible doctor | | | | | |
| The company that created AI | | | | | |
| The patient who agreed to follow AI's directions | | | | | |
